# Disaster Preparedness and Its Associated Factors Among Adults Residing in Urban Slums of Bhubaneswar: A Cross-Sectional Study

**DOI:** 10.7759/cureus.109786

**Published:** 2026-05-27

**Authors:** Krishna Mishra, Vedaant Parekh, Baishnabi Pattanaik

**Affiliations:** 1 Department of Community Medicine, Kalinga Institute of Medical Sciences, Bhubaneswar, IND

**Keywords:** cyclones, disaster, floods, impact, preparedness, urban slums

## Abstract

Introduction

Disasters are unplanned events with the potential to cause disruption to life and property, warranting immediate action. However, preparedness for the same can be beneficial in saving lives and decreasing loss to property. This has graver consequences in urban slums compared to better furnished regions owing to improper housing conditions, low standard of living and improper drainage in the latter. Odisha is a land of many cyclones and floods, and hence, the current study was undertaken to assess the level of disaster preparedness among adults residing in the urban slums of Bhubaneswar and to determine the factors affecting the level of disaster preparedness among them.

Methods

This cross-sectional study was carried out in urban slums under the field practice area of a teaching hospital of Odisha over a period of three months (June-August 2024) after getting institutional ethical clearance. A total of 164 adults of either gender who resided in the slums for over one year and who provided informed consent were included in the study. Written informed consent was obtained if the respondent was literate and left a thumb impression in the presence of a witness from his/her family if the participant was illiterate. Data was collected using a semi-structured and pre-tested questionnaire. Only completed data sets (157) were entered in a Microsoft Excel (Microsoft Corp., Redmond, WA, USA) spreadsheet and analyzed using Epi-Info software version 7.2.5. Chi-square test and f-test, wherever applicable, were used as the tests of significance, and a p-value < 0.05 was considered statistically significant. Odds ratios and confidence intervals were used for better interpretation of the strength of association.

Results

A total of 157 completed data sheets were analyzed. Around 147 (93.63%) of the study participants had faced some kind of disaster (cyclones and/or floods), but only 105 (66.88%) of the entire study population had heard about disaster preparedness. The level of disaster preparedness was assessed using a set of 12 questions, and according to the number of correctly answered questions, the participants were grouped under different categories. Of the total population, only 49 (31.21%) of the participants had moderate preparedness, while the other 108 (68.79%) were somewhat unprepared. It was seen that factors like gender of the respondent (p = 0.022), socio-economic status (p = 0.0019), those who had ever faced a disaster (p = 0.0024) and the kind of disaster faced (if any) (p = < 0.0001) were some of the factors associated with disaster preparedness in the study population.

Conclusion

Disaster preparedness plays an important role in reducing its impact, and the current study depicted that some level of preparedness was present in all the study participants. Some of the socio-demographic factors, like female gender, socio-economic status and having faced a disaster in the past, played a major role in disaster preparedness among the adult residents of the urban slum community.

## Introduction

Disasters are inevitable in most cases, but preparedness for the same can be beneficial in saving lives and decreasing loss to property. The direct impact of a disaster can lead to the disruption of infrastructure and increased risk to the poor. This has harmful consequences in urban slums due to lower housing standards, poor drainage across the area and the presence of potential vector breeding areas in and around the houses. Disasters are often associated with disruption in the supply of clean drinking water and a lack of basic sanitation. Informal settlements like urban slums, which are home to many urban poor, tend to be located in areas most vulnerable to natural hazards such as cyclones, flooding following heavy rains or high tides, strong winds, forest fires, and earthquakes. Conceptualization of the risk profile among the residents of such areas presents a starting point where the evaluation of the impacts due to environmental hazards can be understood and mitigated effectively [1]. As reported by the World Health Organization (WHO), climate change is impacting health in numerous ways, as evident from the reports on morbidities and mortalities from increasingly frequent extreme weather events. The effects are seen when disasters occur in a specified area, like when heatwaves prevail or cyclones and floods hit, there is disruption of food systems and a rise in the cases of zoonosis. There is also a marked surge in food, water and vector-borne diseases. Climate change impacts the social determinants for health directly, thereby affecting livelihoods as well as accessibility and availability of health care. These health risks owing to climate change impact the vulnerable and underserved population that include women and children, minorities from different ethnic groups, the urban poor, migrants, geriatric people, and those with underlying morbidities [2]. The National Disaster Management Authority (NDMA) has developed a web-based Dynamic Composite Risk Atlas and Decision Support System (Web-DCRA & DSS tool) for cyclone risk mitigation and response planning. This recently developed tool has been put to use in a few recent cyclones. The Flood Hazard Atlas has been developed by the National Remote Sensing Centre (NRSC) for flood-prone states of India, including Odisha, where artificial intelligence (AI) is used to forecast, prepare and mitigate upcoming disasters [3].

The impacts of these natural hazards in areas prone to them can be attributed to a lower level of preparedness, thereby increasing the social, physical and economic vulnerability of residents in those areas [1]. Disaster preparedness is a continuous and integrated process involving a wide range of activities and resources rather than just a distinct sectoral activity. Disaster management requires a multi-pronged approach needing support for training of manpower, supply of logistics, prompt governmental stewardship, well-prepared and equipped health care infrastructure and most importantly, involvement of the community through community participation [4]. Places with low housing standards and communities with higher vulnerability need to be aware of the preparedness, which can help curb the morbidities and mortalities during and/or after a disaster [5].

Odisha is a state that is often prone to cyclones, rainfall and consequent floods [6]. The impact of the frequent disasters results in a sharp increase in vector-borne diseases and gastrointestinal illness among the residents of the slums, as evident from the patient turnover in the outpatient departments (OPDs), which are largely preventable. The social and economic impact of disasters can be reduced if people facing them are prepared, with timely forecasts and small steps taken at the household level. Unfortunately, there is limited research about the preparedness of various communities at the household level in this part of Odisha. Hence, the current study was planned to assess the level of disaster preparedness among adults residing in the urban slums of Bhubaneswar and to determine the factors affecting the level of preparedness for common disasters in this area.

The study was presented as an oral paper in the 42nd Annual Conference of IAPSM (OSB) held in Odisha on 13th December, 2024.

## Materials and methods

Study design, population and setting

This cross-sectional study was undertaken in the urban slums under the field practice area of Urban Health Training Centre (UHTC) of a tertiary care and teaching hospital in Bhubaneswar, Odisha. The study commenced for a period of three months (June-August 2024).

Inclusion and exclusion criteria

Adult (>18 years) respondents of either gender from the selected households who gave written informed consent, could comprehend the questions asked, and were not deaf or mute were included in the study. Households that did not have even one adult respondent satisfying the inclusion criteria, uncooperative respondents, bedridden individuals and houses locked even after two visits were excluded from the study.

Sample size calculation

The sample size was calculated to be 164, assuming the prevalence of disaster preparedness level to be 50% (as there is paucity of similar published studies on this population and this area), considering the CI (confidence interval) as 95%, power as 80% and an allowable error of 05%.

Sampling technique

The total population of all five slums under the field practice area of UHTC is around 12,500 with 3200 households. The households to be included in the study were selected by a systematic random sampling technique. Every 20th house was included, and the sampling interval was calculated by dividing the total number of households with the sample size (3200 / 164 = 19.51 ≈ 20). After selection of the households, one eligible study participant was approached according to availability and willingness to participate in the study.

Study tool

An interviewer-administered semi-structured questionnaire was used for data collection (see Supplementary file in Appendices). The questionnaire was prepared after a thorough literature review of published studies and referring to the locally available documents and resources [7-10]. It was validated through inputs from three subject experts, including an emergency medicine physician, one Red Cross member and an officer from the disaster management authority of Odisha. The internal consistency of the questionnaire was assessed by calculating the Cronbach’s α value, which was 0.894. It was pilot-tested in another slum and modified as per requirement, and was translated into the local language while interviewing. The responses were recorded by the investigator or their team member. The questionnaire consisted of three parts for data acquisition on the stated objectives. The first part captured the socio-demographic details of the family, the second part had details of the knowledge on disasters and disaster preparedness, and the third part was to assess the utilization pattern of the available disaster preparedness resources, and included questions to assess the source of information regarding the above points. A set of 12 questions based on disaster preparedness were framed to determine the level of preparedness and it was ranged from ‘unprepared’ if they answered ‘no or incorrect answer or don’t know’ to all the questions, ‘somewhat unprepared’ if they said ‘yes or answered correctly’ to 25%, i.e. three of the questions, ‘moderately prepared’ if they answered ‘yes or answered correctly’ to ≥ 50%, i.e. six of the questions and ‘well prepared’ if they said ‘yes or answered correctly’ to ≥ 75%, i.e. nine of the questions asked. The socio-economic status (SES) of the study participants was assessed using the Modified Kuppuswamy socio-economic scale 2024 [11].

Data collection method

The first household included in the study was the first house enlisted in the family survey and household register that is maintained in the UHTC. Subsequently, each 20th household was considered. Prior permission and appointment were sought from the participant. The household was visited at the pre-decided time for data collection. In case any of the selected households did not fulfill the selection criteria, the next house adjacent to the excluded house was approached.

Data analysis

The data was entered into Microsoft Excel (Microsoft Corp., Redmond, WA, USA) and analyzed using Epi-Info software version 7.2.5. Chi-square test and f-test, wherever applicable, were used, and a p-value < 0.05 was considered statistically significant. The odds ratio was calculated to estimate the strength of association.

Operational definitions

Disaster preparedness was defined operationally as the ability to predict, respond, and cope with the effects of a disaster [12]. A resident was described as 'somewhat unprepared' operationally when he/she did not keep a medical kit ready or did not shift to shelter homes before a disaster or did not procure dry food items beforehand, even if they did other preparations for a disaster. The study participant was operationally defined as 'moderately prepared' when he/she moved household items to a safer location, purchased dry food items, kept an emergency kit ready, participated in disaster response, recovery or training, and shifted to safer locations before a disaster. Permanent residents were those who resided in the slums for more than 10 years, and migrants were defined as those who lived in that slum for less than 10 years and had migrated from elsewhere for work or stay.

Ethical considerations

Clearance from the Institutional Ethics Committee of the institute was obtained via letter no. KIIT/KIMS/1849/2024, and the study was conducted in accordance with the Indian Council of Medical Research (ICMR) ethical guidelines for conducting biomedical research. Written informed consent was obtained from all study participants after explaining the purpose of the study. Confidentiality was maintained throughout the study. Participants were informed in advance that they could leave the interview at any time and skip any questions they did not wish to answer.

## Results

**Figure 1:**
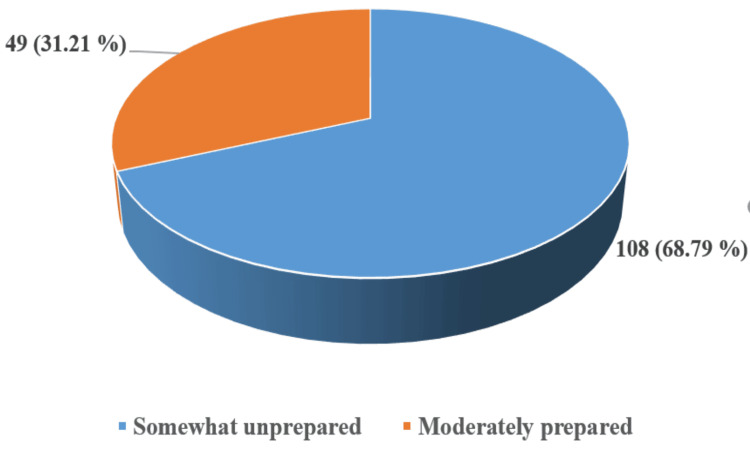
The level of disaster preparedness among the study participants [n (%)]

The calculated sample size was 164, but four respondents refused to give all the required information and withdrew themselves from the study between the interview, and three questionnaires were incompletely filled and hence not analyzed. Hence, a total of 157 data sets were analyzed and included in the study. The mean age of the study participants was 34.28 ± 07.10 years with a range from 27 to 41 years. As per a set of 12 questions regarding various aspects of disaster preparedness, the level of preparedness was assessed. Around 49 (31.21%) of the study participants could answer around half of the questions and hence were ‘moderately prepared’ for facing a disaster at the household level, whereas around 108 (68.79%) belonged to the ‘somewhat unprepared’ category (answered around three questions). There were none who were completely ‘unprepared’ or ‘well prepared’ for a disaster in the future. This preparedness was for cyclones, floods and high waves. None of the study participants responded to have experienced an earthquake or heat stroke or had any preparedness regarding the same. The level of disaster preparedness is depicted in Figure 1.

Table 1 represents the various socio-demographic factors affecting the level of disaster preparedness among the study participants. Crude odds ratio (OR) and confidence intervals were calculated and represented, and bivariate analysis was conducted. Chi-square test and F-test, wherever applicable, were used as tests of significance. The table depicts that there was a statistically significant (p = 0.0220) difference between the level of preparedness among the males and females; females had a better preparedness level. The participants belonging to lower SES had a significantly higher level of preparedness (p = 0.0019) compared to participants from higher SES. Other socio-demographic factors like the family size, type of housing, occupation of the head of the household and the respondent were also assessed, but they were not found to be statistically significant (p < 0.05). Higher odds of preparedness (1.12) were found among permanent residents.

**Table 1:** Association of various sociodemographic variables with the level of disaster preparedness among the study participants (n = 157) Values have been presented as n (%), p-values < 0.05 have been considered as statistically significant, as marked with an *, Ref in the last column refers to the Reference variable.

Socio-demographic variables of the participants	Somewhat unprepared (n = 108) [n (%)]	Moderately prepared (n = 49) [n (%)]	χ ^2^	p-value	OR (95% CI)
Gender					
Male (n=43)	36 (83.72%)	07 (16.28%)	05.23	0.0220*	0.33 (0.13–0.83)
Female (n=114)	72 (63.15%)	42 (36.85%)			1 (Ref)
Religion					
Hindu (n=148)	104 (70.27%)	44 (29.73%)	01.57	0.2101	0.34 (0.08–1.41)
Muslim and others (n=9)	04 (44.45%)	05 (55.55%)			1 (Ref)
Caste					
General (n=70)	49 (70.00%)	21 (30.00%)	00.01	0.9201	0.90 (0.45–1.80)
SC (scheduled caste) and OBC (Other backward classes) (n=87)	59 (67.82%)	28 (32.18%)			1 (Ref)
Residential status					
Permanent (n=103)	70 (67.96%)	33 (32.04%)	00.02	0.8875	1.12 (0.53–2.36)
Migrant (n=54)	38 (70.37%)	16 (29.63%)			1 (Ref)
Socio-economic status (SES) as per Kuppuswamy SES 2024					
Upper (n=41)	34 (82.92%)	07 (17.08%)	17.02	0.0019*	0.20 (0.04-1.04)
Upper middle (n=51)	41 (80.39%)	10 (19.61%)			0.24 (0.05–1.09)
Lower middle (n=31)	16 (51.61%)	15 (48.39%)			0.91 (0.20–4.05)
Upper lower (n=23)	12 (52.17%)	11 (47.83%)			0.77 (0.17–3.49)
Lower (n=11)	05 (45.45%)	06 (54.55%)			1 (Ref)
Educational status					
Illiterate (n=15)	06 (40.00%)	09 (60.00%)	07.37	0.0610	3.00 (0.54–16.60)
Below primary school (n=72)	49 (68.05%)	23 (31.95%)			8.20 (1.32–50.90)
Middle school (n=33)	25 (75.75%)	08 (24.25%)			2.00 (0.34–11.60)
High school (n=37)	28 (75.67%)	09 (24.33%)			1 (Ref)

Table 2 shows the association of variables related to disaster experience with the level of preparedness among the study participants. The following table depicts that those who had ever faced a disaster (n = 147) in the past had 5.83 times higher odds of preparedness than others, and this was found to be statistically significant (p = 0.0172). Preparedness was also significantly higher for cyclones (p = < 0.0001), and those who had faced it within the previous one to two years (n = 98) showed a significantly higher level of preparedness (p = 0.0017) as compared to those who faced it long ago.

**Table 2:** Association of variables related to disaster experience with level of preparedness among the study participants (n = 157) The values have been represented as n (%), p-values < 0.05 have been considered as statistically significant and marked as *, Ref in the last column refers to the reference variable.

Variables related to disasters	Somewhat unprepared (n = 108) [n (%)]	Moderately prepared (n = 49) [n (%)]	χ ^2^	p-value	OR (95% CI)
Ever faced a disaster					
Yes (n=147)	105 (71.42%)	42 (28.58%)	05.68	0.0172*	5.83 (1.44 - 23.62)
No (n=10)	03 (30.00%)	07 (70.00%)			1 (Ref)
Kind of disaster faced (n=147)					
Cyclones (n=19)	04 (21.05%)	15 (78.95%)	27.97	< 0.0001*	13.70 (3.70 – 50.70)
Floods (n=21)	10 (46.61%)	11 (53.39%)			4.02 (1.37 – 11.80)
Cyclone and flooding (n=107)	84 (78.50%)	23 (21.50%)			1 (Ref)
How long ago did you face the disaster? (n=147)					
6-10 years (n=23)	10 (43.47%)	13 (56.53%)	12.77	0.0017*	0.27 (0.11 – 0.70)
3-5 years (n=26)	12 (46.15%)	14 (53.85%)			0.25 (0.10 – 0.62)
1-2 years (n=98)	17 (13.35%)	81 (86.65%)			1 (Ref)

Table 3 depicts the various activities undertaken by the study participants that affect the level of preparedness. About 83 (52.86%) of the study participants had ever discussed disaster preparedness with their family. The level of preparedness was found to be significantly higher among those who kept an emergency medical kit ready for use in case of need (n = 24) as a preparatory measure (p = < 0.0001) with an odds of 17.93. Statistically significant association of the level of preparedness was also found among those who had ever been a part of disaster training, planning or rescue team (n = 22) (p = 0.0050) with an odds of 4.15. Around 101 (64.33%) of the study participants had kept an alternate source of lighting ready as a preparatory measure. Shifting to shelter homes or a safer place before a disaster was a part of better preparedness in around 32 (71.12%) of the study participants. Around 118 (75.16%) of them knew about safe storage of the food items and other utilities and knew the method of disinfecting them after a disaster. Around 146 (93.00%) of them were aware of the UHTC that caters to their area. As few as 19 (12.10%) of them were aware of the diseases that spread after a disaster and the methods to prevent them. Amongst those who were aware, diarrhea and typhoid were considered as the major diseases faced after a disaster.

**Table 3:** Association of various factors affecting preparedness and level of disaster preparedness among the study participants (n=157) The values have been represented as n (%), a p-value < 0.05 was considered statistically significant and marked as *, Ref in the last column refers to the reference variable.

Variables related to disasters	Somewhat unprepared (n = 108) [n (%)]	Moderately prepared (n = 49) [n (%)]	χ ^2^	p-value	OR (95% CI)
Do you discuss disaster preparedness with your family after getting the information?					
Yes (n=83)	58 (69.87%)	25 (30.13%)	0.06	0.8065	0.90 (0.47–1.74)
No (n=74)	50 (67.56%)	24 (32.44%)			1 (Ref)
Do you keep an emergency medical kit ready before a disaster?					
Yes (n=24)	04 (16.66%)	20 (83.34%)	33.04	< 0.0001*	17.93 (5.63–57.10)
No (n=133)	104 (78.20%)	29 (21.80%)			1 (Ref)
Do you keep an alternate source of light handy before a disaster?					
Yes (n=101)	52 (51.48%)	49 (48.52%)	2.30	0.1294	0.56 (0.29–1.08)
No (n=56)	21 (37.50)	35 (62.50%)			1 (Ref)
Do you shift to a shelter home/ relative’s place or a safer place before a disaster?					
Yes (n=45)	13 (28.88%)	32 (71.12%)	1.31	0.2524	3.66 (1.74–7.71)
No (n=112)	67 (59.82%)	45 (40.18%)			1 (Ref)
Do you know what services the Government provides before a disaster?					
Yes (n=09)	08 (88.89%)	01 (11.11%)	0.94	0.3323	0.26 (0.03–2.19)
No (n=148)	100 (67.57%)	48 (32.43%)			1 (Ref)
Does your family know what to do before a cyclone or flood?					
Answered correctly (n=118)	83 (70.34%)	35 (29.66%)	0.28	0.5967	0.75 (0.35–1.62)
Could not answer (n=39)	25 (64.10%)	14 (35.90%)			1 (Ref)
Do you keep away all the things that might come in contact with flood water in a safe place to avoid contamination?					
Yes (n=118)	83 (70.30%)	35 (29.70%)	0.28	0.5967	0.75 (0.35–1.62)
No (n=39)	25 (64.10%)	14 (35.90%)			1 (Ref)
Do you know what diseases spread after a disaster and how you can protect yourself and your family?					
Answered correctly (n=19)	11 (57.89%)	08 (42.11%)	0.69	0.4062	1.73 (0.63–4.78)
Does not know (n= 138)	97 (70.29%)	41 (29.71%)			1 (Ref)
Have you ever been involved in disaster pre-preparedness training/ response and/or recovery?					
Yes (n=22)	07 (31.81%)	15 (68.19%)	7.88	0.0050*	4.15 (1.60–10.80)
No (n=135)	89 (65.93%)	46 (34.07%)			1 (Ref)
Are you aware of UHTC near you?					
Yes (n=146)	99 (67.80%)	47 (32.20%)	0.40	0.5271	0.47 (0.10–2.24)
No (n=11)	09 (81.81%)	02 (18.19%)			1 (Ref)
Do you have a health/life insurance?					
Yes (n=27)	20 (74.07%)	07 (25.93%)	0.18	0.6714	0.73 (0.29–1.91)
No (n=130)	88 (67.69%)	42 (32.31%)			1 (Ref)
Do you feel the Government services are sufficient before or after a disaster?					
Yes (n= 09)	06 (66.67%)	03 (33.33%)	0.10	0.7518	1.04 (0.24–4.41)
No (n= 148)	100 (67.57%)	48 (32.43%)			1 (Ref)

Around 113 (71.97%) of the respondents expressed their interest in attending a free disaster preparedness training if provided. The majority of respondents, 148 (94.26%), considered the available government schemes not sufficient enough during a disaster and wanted a prompt response with more cash benefits during the recovery period for the underserved.

**Figure 2:**
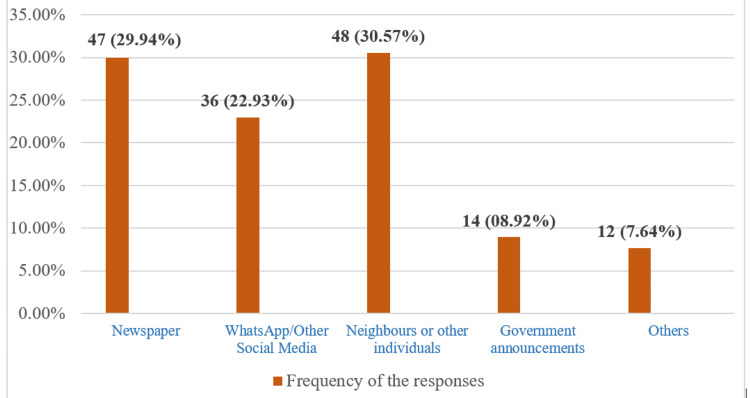
Sources of data regarding disaster preparedness in percentage [n (%)] *Others included multiple sources of receiving information regarding pre-preparedness for a disaster.

The source of information regarding the disasters and preparedness was evaluated and is depicted in Figure 2. A maximum of the study participants, 48 (30.57%), came to know about disaster preparedness from their neighbors or others, followed by information from newspapers, 47 (29.94%). Others came to know from social media, 36 (22.93%), or government announcements, 14 (08.92%), or multiple sources, 12 (07.64%).

## Discussion

The present study was conducted among urban slum residents to assess their disaster preparedness. The mean age of the study participants was 34.28 ± 7.1 years. In a qualitative study done by Jessica Yu et al., housing standards, ownership status of the house, and storage of livelihood goods were identified as some of the indicators of disaster preparedness [5]. Some of these factors were also considered in the current study, but were not found to be statistically significant. The present study depicted that all participants had some kind of preparedness, and around 147 (93.60%) had experienced a disaster. In a study done among university students, around 57 (63.00%) of the students were not aware of any preparedness [13]. They considered that the university should be ready with first-aid kits in case of need. The findings of the current study are different than the cited study. An individual level of preparedness is required in order to decrease the impact at a personal level, in the form of decreasing the inconvenience caused personally.

Odisha is home to many cyclones and subsequent floods. Vector-borne disease outbreaks are common due to the presence of potential vector-breeding sites nearby [14]. However, the current study shows that as few as 19 (12.10%) of the study participants were aware of the diseases that can spread after a disaster and its protective measures. The awareness regarding the diseases resulting from a disaster and the protective measures was found to be low in the study population. Disaster training and frequent health education sessions might be helpful in improving the awareness among the study population and decreasing the burden on the health system. Personal awareness can lead to personal protection and prevention of an outbreak following a disaster, thereby curbing morbidities and mortalities. A study done in South Korea reported that the occupation of the study participants, SES, experience of a disaster beforehand, and knowledge of disaster preparedness were some of the predictors of disaster preparedness at the household level [15]. Some of the factors, like SES, prior disaster experience, type of disaster faced and the gender of the study participants, were found to be associated with disaster preparedness. So, the results of both studies are similar. In another study done in Taiwan, some disaster preparedness behaviors including the purchase of an insurance, keeping an emergency kit ready as a preparatory measure, identifying and planning evacuation locations and routes, and the participation of the respondents in disaster response drills were depicted by less than 47 (11.90%) of the participants, and older adults exhibited less preparedness as compared to the younger ones [16]. The result of the present study depicts a higher level of preparedness with respect to the above four behaviors. In another study done in the United States, factors like ‘discussed disaster preparedness with others’, ‘attending a session on disaster training’, and ‘previously experienced a disaster’ were found to be statistically significantly associated with preparedness [17]. The results of the current study align with the results of the cited study. The current study found a statistically significant association between the level of preparedness and involvement of the resident in disaster response, training or rescue. A study conducted long back in the year 2012 in Itanagar had enforced the importance of training and mock drills as important factors affecting the preparedness to face a disaster [18]. The present study also emphasizes the same. As per these findings, the importance of training sessions should be emphasized as a public health measure for adequate disaster preparedness in a vulnerable mass like slum dwellers.

The source of information regarding disasters and disaster preparedness in the current study was mainly from neighbors, followed by newspapers and social media, whereas a study done in Tamil Nadu showed that newspapers were the primary source of information for the residents [19]. In a study done in Saudi Arab, the most common source of information regarding disaster and preparedness was social media, 572 (78.80%), and despite the majority of the study population showing a positive attitude toward disaster preparedness, only 248 (34.20%) of the respondents were previously involved in a disaster drill(s) or workshop(s) [20]. These results are similar to those of the present study.

This part of the state, being a disaster-prone area, mostly due to cyclones and floods, requires better preparedness among inhabitants, and slums, being areas lacking proper drainage, presence of open drains, poor house infrastructure, overcrowded families, with potential vector-breeding sites and prone to accidents due to flying objects following strong winds, need special emphasis and household training regarding various aspects of disaster preparedness. Policies to ensure the supply of clean piped drinking water that do not easily get destroyed or broken during disasters need to be strengthened. Outbreaks of diseases like typhoid, gastroenteritis, malaria, dengue and parasitic infections can be prevented largely by adequate and frequent training of the inhabitants, thereby enabling them to take proper steps to protect their family and the community at large. Hence, disaster preparedness has its own public health importance.

Limitations

Being cross-sectional in nature, causality cannot be established. The results of the study cannot be generalized as it was conducted among urban slum residents with varied levels of literacy status. The preparedness behaviors were self-reported by participants, and hence, the chances of social desirability bias cannot be completely excluded.

## Conclusions

The present study identified that all participants who were residents of urban slums had some level of disaster preparedness at their household level; some were ‘moderately prepared’ for facing a disaster at the household level, whereas others were ‘somewhat unprepared’. None of the respondents was completely unprepared. There were certain sociodemographic factors that were identified to be associated with the level of disaster preparedness, such as the gender of the participant and the socioeconomic status of the family. An experience of facing a disaster in the recent past, type of disaster faced (if any) by the resident, keeping an emergency kit ready before a disaster and the involvement of the resident in the preparedness/rescue or recovery phase were some other factors found to be associated with the level of disaster preparedness. These factors show that training on disaster preparedness might be an important step to increase preparedness level among the study population, as many of them were willing to be a part of the training program.

Frequent training regarding the various aspects of disaster preparedness to improve community resilience through community participation is the need of the hour. Household and individual protection and prevention of health hazards during or after a disaster can result in an economically independent community, decreasing the pressure on the existing health facilities. Strengthening the existing policies might be considered with the inclusion of scope for training of the inhabitants, the service providers and healthcare providers. The involvement of community volunteers might be helpful in building a stronger and well-prepared community to face a disaster in case of need.

## Appendices

**Table 4:** Supplementary file: Questionnaire

Section	Variable / Question	Response Options / Coding
PART 1: Socio-Demographic Details	Name of respondent	Open-ended
	Date of interview	Open-ended
	Head of household (HH)?	1 = Yes, 2 = No
	Contact number	Open-ended
	Name of head of HH	Open-ended
	Age of respondent	Numeric
	Gender	1 = Male, 2 = Female, 3 = Others
	Religion	1 = Hindu, 2 = Muslim, 3 = Sikh, 4 = Christian, 5 = Others
	Caste	1 = General, 2 = SC, 3 = ST, 4 = OBC
	Residential status	1 = Permanent (>1 year), 2 = Migrant
	Education of respondent	1 = Illiterate, 2 = Below primary school, 3 = Middle school, 4 = High school, 5 = Intermediate/Post high school diploma, 6 = Graduate/PG, 7 = Professional/Honors
	Highest education of HH	1 = Illiterate, 2 = Below primary school, 3 = Middle school, 4 = High school, 5 = Intermediate/Post high school diploma, 6 = Graduate/PG, 7 = Professional/Honors
	Employment status of head of HH	1 = Unemployed, 2 = Street Vendor/Small Informal Business, 3 = Government Employee, 4 = Own Business, 5 = Private Employee, 6 = Other
	Occupation of respondent	1 = Unemployed, 2 = Street Vendor/Small Informal Business, 3 = Government Employee, 4 = Own Business, 5 = Private Employee, 6 = Other
	SES as per Kuppuswamy 2024	1 = Upper, 2 = Upper Middle, 3 = Lower Middle, 4 = Upper Lower, 5 = Lower
	Family size	Numeric
	Type of family	Open-ended
	Type of house	1 = Pucca, 2 = Kutcha, 3 = Semi-pucca
PART B: Knowledge and Practice During a Disaster	Community recently experienced disaster?	1 = Yes, 2 = No
	If yes, how many years ago	1 = 6–10 years ago, 2 = 3–5 years ago, 3 = 1–2 years ago
	Type of disaster experienced	Multiple response: 1 = Drought, 2 = Wildfire, 3 = Flooding, 4 = Cyclones, 5 = Landslide, 6 = Earthquake, 7 = Tornado/Winds, 8 = Industrial Accidents, 9 = Cyclones & Flooding together, 10 = Others
	Preparedness level	1 = Not prepared, 2 = Somewhat unprepared, 3 = Moderately prepared, 4 = Well prepared, 5 = Very well prepared
	Discuss disaster preparedness with family	1 = Yes, 2 = No
	Emergency medical kit available	1 = Yes, 2 = No
	Alternate source of light available	1 = Yes, 2 = No
	Life insurance available	1 = Yes, 2 = No
	Awareness of UHTC nearby	1 = Yes, 2 = No
	Involved in disaster preparedness/response/recovery	1 = Yes, 2 = No
	Action during disaster	Multiple response: 1 = Evacuate, 2 = Remain inside house, 3 = Continue daily activities, 4 = Follow public/government instructions, 5 = Others
	Family members know electrical shutdown during floods/cyclones	1 = Yes, 2 = No
	Keep away/discard flood-contaminated goods	1 = Yes, 2 = No
	Backup source for shelter/food/water	1 = Yes, 2 = No
	Shift to shelter homes during disaster	1 = Yes, 2 = No
	Awareness of government disaster management schemes	1 = Yes, 2 = No
PART 3: Utilization of Available Services	Existing management schemes sufficient?	1 = Yes, 2 = No, 3 = Not sure
	Community helpful during disaster	1 = Yes, 2 = No
	Changes desired in existing schemes	Open-ended
	Open to free disaster preparedness training	1 = Yes, 2 = No, 3 = Not sure
	Receive cooked/dry food during rescue period	1 = Yes, 2 = No, 3 = Not applicable
	Time taken to return home after disaster	Open-ended
	Aware of diseases spreading after disaster	1 = Yes, 2 = No
	Diseases aware of	Open-ended
	Educated about prevention of such diseases	1 = Yes, 2 = No
	Received vaccines after disaster	1 = Yes, 2 = No
	Source of preparedness information	Multiple response: 1 = Newspaper, 2 = WhatsApp/Social Media, 3 = Neighbours/Others, 4 = Government announcements, 5 = Others
	Source provides all required information	1 = Yes, 2 = No
	Trusting social media information is right	1 = Yes, 2 = No
Section	Variable / Question	Response Options / Coding
PART 1: Socio-Demographic Details	Name of respondent	Open-ended
	Date of interview	Open-ended
	Head of household (HH)?	1 = Yes, 2 = No
	Contact number	Open-ended
	Name of head of HH	Open-ended
	Age of respondent	Numeric
	Gender	1 = Male, 2 = Female, 3 = Others
	Religion	1 = Hindu, 2 = Muslim, 3 = Sikh, 4 = Christian, 5 = Others
	Caste	1 = General, 2 = SC, 3 = ST, 4 = OBC
	Residential status	1 = Permanent (>1 year), 2 = Migrant
	Education of respondent	1 = Illiterate, 2 = Below primary school, 3 = Middle school, 4 = High school, 5 = Intermediate/Post high school diploma, 6 = Graduate/PG, 7 = Professional/Honors
	Highest education of HH	1 = Illiterate, 2 = Below primary school, 3 = Middle school, 4 = High school, 5 = Intermediate/Post high school diploma, 6 = Graduate/PG, 7 = Professional/Honors
	Employment status of head of HH	1 = Unemployed, 2 = Street Vendor/Small Informal Business, 3 = Government Employee, 4 = Own Business, 5 = Private Employee, 6 = Other
	Occupation of respondent	1 = Unemployed, 2 = Street Vendor/Small Informal Business, 3 = Government Employee, 4 = Own Business, 5 = Private Employee, 6 = Other
	SES as per Kuppuswamy 2024	1 = Upper, 2 = Upper Middle, 3 = Lower Middle, 4 = Upper Lower, 5 = Lower
	Family size	Numeric
	Type of family	Open-ended
	Type of house	1 = Pucca, 2 = Kutcha, 3 = Semi-pucca
PART B: Knowledge and Practice During a Disaster	Community recently experienced disaster?	1 = Yes, 2 = No
	If yes, how many years ago	1 = 6–10 years ago, 2 = 3–5 years ago, 3 = 1–2 years ago
	Type of disaster experienced	Multiple response: 1 = Drought, 2 = Wildfire, 3 = Flooding, 4 = Cyclones, 5 = Landslide, 6 = Earthquake, 7 = Tornado/Winds, 8 = Industrial Accidents, 9 = Cyclones & Flooding together, 10 = Others
	Preparedness level	1 = Not prepared, 2 = Somewhat unprepared, 3 = Moderately prepared, 4 = Well prepared, 5 = Very well prepared
	Discuss disaster preparedness with family	1 = Yes, 2 = No
	Emergency medical kit available	1 = Yes, 2 = No
	Alternate source of light available	1 = Yes, 2 = No
	Life insurance available	1 = Yes, 2 = No
	Awareness of UHTC nearby	1 = Yes, 2 = No
	Involved in disaster preparedness/response/recovery	1 = Yes, 2 = No
	Action during disaster	Multiple response: 1 = Evacuate, 2 = Remain inside house, 3 = Continue daily activities, 4 = Follow public/government instructions, 5 = Others
	Family members know electrical shutdown during floods/cyclones	1 = Yes, 2 = No
	Keep away/discard flood-contaminated goods	1 = Yes, 2 = No
	Backup source for shelter/food/water	1 = Yes, 2 = No
	Shift to shelter homes during disaster	1 = Yes, 2 = No
	Awareness of government disaster management schemes	1 = Yes, 2 = No
PART 3: Utilization of Available Services	Existing management schemes sufficient?	1 = Yes, 2 = No, 3 = Not sure
	Community helpful during disaster	1 = Yes, 2 = No
	Changes desired in existing schemes	Open-ended
	Open to free disaster preparedness training	1 = Yes, 2 = No, 3 = Not sure
	Receive cooked/dry food during rescue period	1 = Yes, 2 = No, 3 = Not applicable
	Time taken to return home after disaster	Open-ended
	Aware of diseases spreading after disaster	1 = Yes, 2 = No
	Diseases aware of	Open-ended
	Educated about prevention of such diseases	1 = Yes, 2 = No
	Received vaccines after disaster	1 = Yes, 2 = No
	Source of preparedness information	Multiple response: 1 = Newspaper, 2 = WhatsApp/Social Media, 3 = Neighbours/Others, 4 = Government announcements, 5 = Others
	Source provides all required information	1 = Yes, 2 = No
	Trusting social media information is right	1 = Yes, 2 = No
